# Sexually Transmitted Infections and Associated Factors in Southeast Spain: A Retrospective Study from 2000 to 2014

**DOI:** 10.3390/ijerph17207449

**Published:** 2020-10-13

**Authors:** María Ángeles Pérez-Morente, María Gázquez-López, María Adelaida Álvarez-Serrano, Encarnación Martínez-García, Pedro Femia-Marzo, María Dolores Pozo-Cano, Adelina Martín-Salvador

**Affiliations:** 1 Department of Nursing, Faculty of Health Sciences, University of Jaén, 23071 Jaén, Spain; mmorente@ujaen.es; 2 Department of Nursing, Faculty of Health Sciences, University of Granada, 51001 Ceuta, Spain; mgazquez@ugr.es; 3 Department of Nursing, Faculty of Health Sciences, University of Granada, 18016 Granada, Spain; pozocano@ugr.es; 4 Guadix High Resolution Hospital, 18500 Guadix, Granada, Spain; 5 Department of Statistics, Faculty of Health Sciences, University of Granada, 18016 Granada, Spain; pfemia@ugr.es; 6 Department of Nursing, Faculty of Health Sciences, University of Granada, 52005 Melilla, Spain; ademartin@ugr.es

**Keywords:** sexually transmitted diseases, public health, risk groups

## Abstract

The World Health Organization estimates that more than one million people acquire a Sexually Transmitted Infection (STI) every day, compromising quality of life, sexual and reproductive health, and the health of newborns and children. It is an objective of this study to identify the factors related to a Sexually Transmitted Infection diagnosis in the province of Granada (Spain), as well as those better predicting the risk of acquiring such infections. In this study, 678 cases were analyzed on a retrospective basis, which were treated at the Centre for Sexually Transmitted Diseases and Sexual Orientation in Granada, between 2000–2014. Descriptive statistics were applied, and by means of binary logistic regression, employing the forward stepwise-likelihood ratio, a predictive model was estimated for the risk of acquiring an STI. Sex, age, occupation, economic crisis period, drug use, number of days in which no condoms were used, number of sexual partners in the last month and in the last year, and number of subsequent visits and new subsequent episodes were associated with an STI diagnosis (*p* < 0.05). The risk of being diagnosed with an STI increased during the economic crisis period (OR: 1.88; 95%-CI: 1.28–2.76); during the economic crisis and if they were women (OR:2.35, 95%- CI: 1.24–4.44); and if they were women and immigrants (OR: 2.09; 95%- CI:1.22–3.57), while it decreased with age (OR: 0.97, 95%-CI: 0.95–0.98). Identification of the group comprised of immigrant women as an especially vulnerable group regarding the acquisition of an STI in our province reflects the need to incorporate the gender perspective into preventive strategies and STI primary health care.

## 1. Introduction

Sexually Transmitted Infections (STIs) constitute a significant public health issue on a worldwide basis. The World Health Organization (WHO) estimates that more than one million people acquire an STI every day, compromising quality of life, sexual and reproductive health, and newborns’ and children’s health [1].

Such infections are caused by more than 30 different bacteria, viruses, and parasites, the most frequent being syphilis, gonorrhea, chlamydia, human papillomavirus (HPV), hepatitis B and C, and human immunodeficiency virus (HIV), the first three of which being curable and the last four being incurable viral infections, although treatments exist to attenuate or modify symptoms and the disease [1].

STIs are more frequent in people with risky sexual behaviors, such as not using condoms, having multiple sexual partners, having a highly risky partner (individual with many sexual partners or other risk factors), having anal sex or a partner who practices it, or having sexual intercourse with a partner who injects or has injected drugs before [2,3,4]. Teenagers, immigrants, sex workers, men who have sex with men (MSM), and bisexual individuals are particularly vulnerable groups regarding the acquisition of STIs [5,6].

Furthermore, the current financial situation impacts on the incidence of STIs, mainly in the most vulnerable population [1,7,8,9,10]. In the recent economic crisis period (2008–2014) [11,12,13,14], syphilis and gonorrhoea reappeared in Spain, and the incidence of HIV, hepatitis, and HPV increased. During this period, the probability of the appearance of an STI was higher than in the non-crisis period [15], due to the financial difficulties to access contraceptive methods, thus increasing the absence of protection against STIs [15,16,17].

At the world level, the prevalence of STIs in men and women is similar, with some regional differences. Nevertheless, complications in these processes do affect disproportionately women and generate a strong impact, mainly on their sexual and reproductive health. The WHO points out that the lack of data on STIs at the local level and, in particular, of data classified by sex, compromises the solution to this problem [18]. Moreover, today, despite the outstanding advance in medical knowledge and development of primary health care, sexual prevention programs seem to continue being inefficient, since the number of STIs continues to increase [1]. In this sense, an increase in STIs may be observed in Spain in general [19] and in the province of Granada in particular [10,15], which becomes an especially alarming issue.

The objective of this study was to identify any factors associated with STI diagnoses in the province of Granada (Spain), as well as those that better predict the risk of acquiring such infections.

## 2. Materials and Methods

The group of cases involving users attended at the Center for Sexually Transmitted Diseases and Sexual Orientation in Granada (Spain) with a new STI diagnosis from 2000–2014 was analyzed on a retrospective basis. This center is attached to the Andalusian Health Service (AHS), which offers universal and free healthcare to people residing in the province regardless of their nationality and income level, providing healthcare to 730,000 people older than 15 years old [20].

The main source of information was the patient´s medical history in which health professionals completed sociodemographic data, symptoms, and medicals signs, results of diagnostic tests, therapeutic evolution, and final diagnosis. The study population consists of users with a positive or negative STI diagnosis clearly recorded on the medical histories contained in a database of 1437 histories created in a previous project from which this study is derived, and whose details on the sampling process are described in another paper [15]. Exclusion criteria included being younger than 18 years old and having a cognitive deficit. Investigators collected the information and attended a training course to reduce possible variations among them.

STI diagnosis was included as the dependent variable and coded as binary (yes/no), following the pattern established by other studies in this line of research [21]. Independent variables were classified in three groups: 1) Sociodemographic characteristics such as sex (female/male), age, nationality (Spanish, foreigner), occupation (sex worker/former worker/others), working status (active/non-active), education level (with no studies or primary/secondary/higher-level education); crisis period, with the year 2008 being considered the commencement of the financial crisis in Spain [11,12,13,14] (yes/no); 2) characteristics regarding the healthcare received, such as reason for consultation (HIV/another); previous treatment (yes/no), number of subsequent consultations, number of new subsequent episodes, and 3) STI risk indicators such as sexual behaviour (heterosexual, homosexual, bisexual); stable relationship (yes/no); stable relationship with symptoms (yes/no); days elapsed since the last contact without using a condom; number of partners in the last month; number of partners in the last year; sex life understood as the total number of partners in their whole life; drug use (yes/no); previous STI (yes/no) and age of first sexual intercourse. Information was recorded on an ad hoc designed computerized data collection sheet, which was then exported to the statistical program for analysis.

Variables were analyzed on a descriptive basis calculating measures of central tendency (mean and standard deviation) for quantitative variables and absolute and relative frequencies for categorical variables. Comparison among groups was carried out using the chi-square test and the *t*-test according to the variable nature. The level of statistical significance was established at 0.05. 

Finally, through binary logistic regression, the optimal model that best predicted a positive STI diagnosis was selected, employing the forward stepwise-likelihood ratio. At first, a model with no explanatory variable (only the constant) was used, and, in every step, a variable including a minor *p* was introduced. The potential predictors group comprised first-order interactions among explanatory variables. For each variable included in the model, the odds ratio (OR) and its confidence interval (CI) were calculated at 95%. The validity of the model was verified by means of the Hosmer-Lemeshow goodness-of-fit test. Analyses were performed using the Statistical Package for the Social Sciences (SPSS) program, version 24, (IBM, New York, NY, USA).

Before this study was carried out, approval was obtained from the Biomedical Research Ethics Committee of the province of Granada and from the Management Directorate of the Granada-Metropolitan Health District, which is responsible for the STI clinic where the research was approved. Patient data were handled with the utmost confidentiality and in compliance with the Spanish Organic Law 15/1999, of December 13, on Personal Data Protection, and the Spanish Organic Law 3/2018, of December 5, on Personal Data Protection and a guarantee of digital rights.

## 3. Results

Inclusion criteria were fulfilled by 678 cases, out of which 440 (65.6%) had a positive STI diagnosis and 230 (34.2%), a negative one. Results show that STIs spread homogeneously across the population in terms of nationality, working status, and education level, establishing, with statistically significant differences, that persons diagnosed with an STI are younger (*p* < 0.001), mostly men (*p* < 0.001), who have not been or are not sex workers (*p* < 0.001), and who were diagnosed during the economic crisis period (2008–2014) (*p* < 0.001) (Table 1).

With respect to the healthcare received, differences were found in the number of subsequent visits (*p* = 0.001) and of new subsequent episodes (*p* < 0.001), being more frequent in cases diagnosed with STIs (Table 2).

As regards risk indicators, the group diagnosed with STIs used drugs more frequently, mentioned a higher number of days elapsed since the last unprotected sexual intercourse, and had more sexual partners in the last month and in the last year (Table 3).

Table 4 shows the logistic regression model adjusted that better predicts a positive STI diagnosis. It is noted that the probability of acquiring an STI is higher in a crisis period, if it is to be a woman in the said period, and if it is to be a woman and an immigrant. In addition, this probability diminishes drastically with age (Table 4).

## 4. Discussion

In relation to the sociodemographic characteristics of the sample analyzed, it is noted that the mean age of individuals diagnosed with an STI was lower than in the case of those who did not have them. This result coincides with the publications in the scientific literature, considering the young population as one of the groups most exposed to STIs [22,23,24] along with other vulnerable groups such as gay men [5,25,26,27,28], men who have sex with men (MSM), transgender people, injecting drug users, women, sex workers, and immigrants, especially irregular immigrants [18,29,30,31,32,33]. Likewise, in a recent study on HIV and other STI epidemiology, it is observed that in 2006 the higher number of cases of some STIs such as syphilis, gonorrhea, chlamydia trachomatis, and lymphogranuloma venereum occurred in young adults between 25–44 years old [34], the age range in which the mean age of persons positively diagnosed with STIs is found in our study.

We have found no relationship between sex work and a positive STI diagnosis, despite the already-known vulnerability of such a group [35,36,37]. Although sexual orientation plays a relevant role, gay men sex workers assume more risky practices compared to heterosexuals [38], they were not well represented in our sample. In addition, safe sexual practices are often more present in commercial sexual relations, while they are relaxed in non-commercial sexual relations [38,39], which may have an impact on this study. However, our results coincide with the ones published regarding Spain by other authors, who found a low seroprevalence of some STIs [40], such as HIV, among sex workers [41,42,43]. 

The association found between the period of economic crisis and the increased risk of being diagnosed with STIs is in line with the fact that a crisis debilitates educational and health systems as well as prevention and promotion measures related to sexual health [44]. Therefore, such a situation may lead to an increase in the prevalence of infectious diseases like STIs, in particular to an increase in new HIV diagnoses [45,46]. 

Upon analyzing the healthcare received, the significant association found between the number of subsequent visits and of new episodes, among people who were positively diagnosed with an STI, could be justified by the need for a more intense follow-up of such new diagnoses.

In relation to risk indicators, we have found that drug use becomes a risky practice for transmission of STIs, due to the limitation imposed on the individual’s decision-making capability, making them more vulnerable, as pointed out by other authors [33,47,48]. Evidence associates the low use of condoms with a higher risk of STI contagion [49,50,51,52], in line with our results, despite some nuances. We have analyzed the time period elapsed since the last time a condom was used and not its frequency of use, but literature identifies both factors as strongly associated with a significant risk of acquiring an STI [22,24,53]. Moreover, upon evaluating jointly two of the abovementioned variables, namely, the time elapsed since the last sexual intercourse without using a condom and the economic crisis period, it is observed that, in Spain, as from 2007, there has been a decrease in the use of contraceptive methods and STI prevention measures in one-fifth of casual or sporadic relationships [45]. Therefore, it should be noted that, in the crisis period, financial problems pose obstacles to access to contraception [16], with the consequent risk not only of unintended pregnancy but also of contagion of venereal diseases. Regarding the number of sexual partners in the last month and in the last year, people having a positive diagnosis had more partners, which finding is in line with the literature consulted [22,31].

In the predictive model designed for the diagnosis of STIs in our province, the variables of the economic crisis period and interactions between crisis and women, and between women and non-Spanish nationality remained, regardless of the other variables, showing an increase in risk, while age acted as a protective factor (Table 4). That is, structural determinants of health inequalities are the ones that have had a direct impact on positive STI diagnoses, reinforcing the knowledge that health is not just an individual issue, but it depends, to a great extent, on the surrounding environment [54,55]. 

As previously stated, this study is within the group of studies finding an association between an adverse financial context and bad health results, which becomes worse in women. The fact that women suffer in a different way than men do, the impacts of every financial crisis, has been widely reported [56,57,58,59,60,61]. And this is so because of the different and unequal opportunities men and women have regarding access to powerful, prestigious positions and to available resources, where women are usually at a disadvantage, even in developed countries like ours [62]. Furthermore, it should be pointed out that gender inequality occurring in the context of relationships affects the sexual and reproductive health of women, exposing them to a higher number of risks [45]. 

If to the fact of being a woman, the fact of being a foreigner is added, the likelihood of being diagnosed with a STI in our study increased even more. This result confirms the findings in previous Spanish studies where immigrant women were more exposed to HIV infection [32,33]. In this sense, the last report on Epidemiologic Surveillance of HIV and AIDS in Spain states that among the foreign population with new HIV diagnoses, 56% were women, had a worse immunological response to antiretroviral therapy, showed less follow-up, and less time for therapeutic failure [63]. Although the fact of being a foreigner, especially if coming from low-income countries, usually entails some disadvantages in the destination country, in financial, working, administrative and legal terms, that affect their health in the medium and long term, the austerity and exclusion policies carried out during the economic crisis seem to have sped up the process [64,65], particularly in relation to women’s sexual health. 

Finally, the predictive model results confirm the protective effect of age in relation to the appearance of STIs. The fact that only a small group of European young people state they have access to information on STI prevention, while most of them have erroneous concepts on it and are in favor of causal sexual intercourse and with multiple partners, shows the existing lack of knowledge on these issues and the underestimation of the risk of acquiring STIs to which they are exposed [22].

### Limitations

This study presents some limitations and strengths. On the one hand, due to its cross-sectional nature, associations detected shall not be interpreted as causal relations, but, in any case, they allow for hypotheses that shall require confirmation in subsequent research with more complex designs. It should also be taken into account that the sample of the population analyzed comes from a single center and may not be fully representative of subjects vulnerable to an STI diagnosis in the province, which affects the external validity of the study. Specifically, it is fairly likely that the immigrant population is overrepresented, since, for instance, the native population may have more access to private healthcare for the treatment of these infections, as it occurs with other health/disease processes [66]. Such a fact would minimize the magnitude of the associations found. Nevertheless, the WHO recommendations are in line with the promotion of knowledge at the local level in order to address STIs, which justifies the need for this type of analysis [18]. On the other hand, although we may think people coming to the center may have had risky sexual behavior, information taken from clinical interviews in-person may be influenced by a social desirability bias, which, even in such a case, would not be discriminatory among the groups analyzed. 

Finally, we believe the power of the study to evaluate factors related to STI diagnosis is high for several reasons. A long time period was evaluated; cases with an accurate (yes/no) STI diagnosis were selected, and individual and clinical data taken from medical histories were analyzed which, to the best of our knowledge, contribute more information and of better quality than any population database available in our context.

## 5. Conclusions

Our findings have identified immigrant women as a risk group regarding the acquisition of STIs in the province of Granada, and especially during an economic crisis. However, there is an evident need for further research, with a gender-oriented perspective, of sexual behavior within such a group, as well as any potential limitations to access the health system, in order to understand differences in behavior and, therefore, in health results between men and women.

Public health policies should be aimed at a re-evaluation of prevention strategies present in our province, as well as of services available, reduced during the crisis. Designing and implementing specific, more effective preventive measures that shall prepare immigrant women to face these infections, as well as detecting non-reported cases and controlling and treating existing cases at a sustainable human and economic cost, entail the first-level challenge in the context of STIs. 

## Figures and Tables

**Table 1:** STI diagnosis vs. sociodemographic characteristics.

Variables	Negative STI Diagnosis		Positive STI Diagnosis		*p*
	*n*	Mean (SD)	*n*	Mean (SD)	
Age (*n* = 678)	234	31.21 (10.62)	444	28.16 (8.27)	<0.001
	***n***	***%***	***n***	***%***	
Sex (*n* = 678)					
Female	76	32.5%	213	48%	<0.001
Male	158	67.5%	231	52%	
Nationality (*n* = 663)					
Spanish	178	78.8%	326	74.6%	0.234
Non-Spanish	48	21.2%	111	25.4%	
Occupation (*n* = 622)					
Sex worker/Former sex worker	13	6.4%	72	17.2%	<0.001
Other occupations	191	93.6%	346	82.8%	
Employment status (*n* = 607)					
Employed	97	46.4%	204	51.3%	0.257
Unemployed	112	53.6%	194	48.7%	
Level of education (*n* = 632)					
No education/Primary education	43	19.8%	77	18.6%	0.926
Secondary education	72	33.2%	141	34%	
Higher education	102	47%	197	47.5%	
Economic crisis (*n* = 678)					
Yes (2008–2014)	80	34.2%	243	54.7%	<0.001
No (2000–2007)	154	65.8%	201	45.3%	

Note: STI—Sexually Transmitted Infection; *n*—sample size; SD—standard deviation; *p—p* value.

**Table 2:** STI diagnosis vs. healthcare received.

Variables	Negative STI Diagnosis		Positive STI Diagnosis		*p*
	*n*	%	*n*	%	
Reason for visit (*n* = 678)					
HIV	67	28.7%	151	34%	0.156
Other reasons	167	71.3%	293	66%	
Previous treatment (*n* = 456)					
Yes	53	40.8%	107	32.8%	0.108
No	77	59.2%	219	67.2%	
	***n***	**Mean (SD)**	***n***	**Mean (SD)**	
No. of subsequent visits (*n* = 667)	229	0.95 (1.01)	438	1.30 (1.41)	0.001
No. of new subsequent episodes (*n* = 668)	229	0.34 (0.67)	439	0.79 (1.24)	<0.001

Note: *n*—sample size; SD—standard deviation; *p*—*p* value.

**Table 3:** STI diagnosis vs. risk indicators.

Variables	Negative STI Diagnosis				Positive STI Diagnosis				*p*
	*n*	%			*n*	%			
Sexual orientation identity (*n* = 649)									0.250
Heterosexual	190	85.6			351	82.2			
Bisexual	11	5			17	4			
Homosexual	21	9.5			59	13.8			
Regular partner (*n* = 627)									
Yes	141	66.8			282	67.8			0.808
No	70	33.2			134	32.2			
Regular partner having symptoms (*n* = 242)									
Yes	32	42.1			68	41			0.867
No	44	57.9			98	59			
Drug use (*n* = 284)									
Yes	24	26.4			78	40.4			0.021
No	67	73.6			115	59.6			
Previous STIs (*n* = 534)									0.415
Yes	40	22.2			90	25.4			
No	140	77.8			264	74.6			
	***n***			**Mean (SD)**	***n***			**Mean (SD)**	***p***
No. of days since last sexual contact without a condom (*n* = 382)	123			2.71 (0.87)	259		3.29 (0.83)		0.001
No. of partners in the last month (*n* = 629)	209		1.40 (1.01)		420		1.87 (1.49)		<0.001
No. of partners in the last year (*n* = 626)	208		2.33 (1.6)		418		3.05 (2.06)		<0.001
Sex life (*n* = 114)	45		1.84 (0.90)		99		1.97 (0.88)		0.436
Age of first sexual intercourse (*n* = 318)	86		18.23 (2.9)		232		17.49 (3.1)		0.054

Note: *n*—sample size; SD—standard deviation; *p*—*p* value.

**Table 4:** Logistic regression for STI diagnosis ^†^.

Variables (Reference Category or Units)	OR	(95% CI)
Crisis (yes)	1.88	(1.28–2.76)
Crisis (yes) × Sex (women)	2.35	(1.24–4.44)
Sex (women) × Nationality (Non-Spanish)	2.09	(1.22–3.57)
Age (years)	0.97	(0.95–0.98)

Note: ^†^—Hosmer-Lemeshow goodness-of-fit test: χ^2^ (8) = 3.89; *p* = 0.867. OR—odds ratio; CI: confidence interval.
